# Effective Organs-at-Risk Dose Sparing in Volumetric Modulated Arc Therapy Using a Half-Beam Technique in Whole Pelvic Irradiation

**DOI:** 10.3389/fonc.2021.611469

**Published:** 2021-08-18

**Authors:** Hyunsoo Jang, Jiyeon Park, Mark Artz, Yawei Zhang, Jacob C. Ricci, Soon Huh, Perry B. Johnson, Mi-Hwa Kim, Mison Chun, Young-Taek Oh, O Kyu Noh, Hae-Jin Park

**Affiliations:** ^1^Department of Radiation Oncology, Dongguk University College of Medicine, Gyeongju, South Korea; ^2^Department of Radiation Oncology, University of Florida College of Medicine, Gainesville, FL, United States; ^3^University of Florida Health Proton Therapy Institute, Jacksonville, FL, United States; ^4^Department of Radiation Oncology, Orlando Health Cancer Institute, Orlando, FL, United States; ^5^Department of Radiation Oncology, Ajou University School of Medicine, Suwon, South Korea

**Keywords:** volumetric modulated arc therapy, half beams, whole pelvic conformal radiotherapy, normal tissue complication probability (NTCP), modulation complexity score, dose conformity

## Abstract

**Background:**

Although there are some controversies regarding whole pelvic radiation therapy (WPRT) due to its gastrointestinal and hematologic toxicities, it is considered for patients with gynecological, rectal, and prostate cancer. To effectively spare organs-at-risk (OAR) doses using multi-leaf collimator (MLC)’s optimal segments, potential dosimetric benefits in volumetric modulated arc therapy (VMAT) using a half-beam technique (HF) were investigated for WPRT.

**Methods:**

While the size of a fully opened field (FF) was decided to entirely include a planning target volume in all beam’s eye view across arc angles, the HF was designed to use half the FF from the isocenter for dose optimization. The left or the right half of the FF was alternatively opened in VMAT-HF using a pair of arcs rotating clockwise and counterclockwise. Dosimetric benefits of VMAT-HF, presented with dose conformity, homogeneity, and dose–volume parameters in terms of modulation complex score, were compared to VMAT optimized using the FF (VMAT-FF). Consequent normal tissue complication probability (NTCP) by reducing the irradiated volumes was evaluated as well as dose–volume parameters with statistical analysis for OAR. Moreover, beam-on time and MLC position precision were analyzed with log files to assess plan deliverability and clinical applicability of VMAT-HF as compared to VMAT-FF.

**Results:**

While VMAT-HF used 60%–70% less intensity modulation complexity than VMAT-FF, it showed superior dose conformity. The small intestine and colon in VMAT-HF showed a noticeable reduction in the irradiated volumes of up to 35% and 15%, respectively, at an intermediate dose of 20–45 Gy. The small intestine showed statistically significant dose sparing at the volumes that received a dose from 15 to 45 Gy. Such a dose reduction for the small intestine and colon in VMAT-HF presented a significant NTCP reduction from that in VMAT-FF. Without sacrificing the beam delivery efficiency, VMAT-HF achieved effective OAR dose reduction in dose–volume histograms.

**Conclusions:**

VMAT-HF led to deliver conformal doses with effective gastrointestinal-OAR dose sparing despite using less modulation complexity. The dose of VMAT-HF was delivered with the same beam-on time with VMAT-FF but precise MLC leaf motions. The VMAT-HF potentially can play a valuable role in reducing OAR toxicities associated with WPRT.

## Introduction

Elective whole pelvic radiation therapy (WPRT) irradiating pelvic lymph nodes is regarded as a standard treatment regimen for intermediate- or high-risk rectal, anal, and gynecological cancers (1–3). Clinical outcome studies reported mild acute and late gastrointestinal (GI), genitourinary (GU), and hematological toxicity profiles in anal cancer patients in intensity-modulated radiation therapy (IMRT), employing simultaneously integrated boost technique and image guidance in IMRT for WPRT (3, 4). In addition, IMRT optimized to spare dose to pelvic bone marrow showed dosimetric benefits to reduce GI complications and hematological toxicities in anal and cervical cancer patients in WPRT (5, 6). Although the benefits of WPRT in patients with locally advanced prostate cancer have been controversial (7, 8), its role and gain have been reexamined considering the interaction between radiation therapy (RT) and adjuvant androgen deprivation therapy based on the duration and timing of hormone therapy and field size effect of RT (9). As per a new protocol, recent results also reported treatment gains in short-term androgen deprivation therapy plus pelvic lymph node irradiation in IMRT for prostate cancer (10–12).

However, late and acute GI toxicities have been significant concerns due to extensive fields (13–15), although WPRT can have clinical merits combined with other treatment schemes and a clever dose optimization technique for various treatment sites (16–20). When a large field size has to be used to cover target volumes plus regional lymph nodes in WPRT, organs-at-risk (OAR) exposure is unavoidable as long as the lower abdomen has to be included in the treatment fields. However, if OAR dose can be effectively spared using dose optimization and precise dose delivery using a multi-leaf collimator (MLC), the extended role of RT can be expected for a better outcome in cancer treatment. OAR dose sparing can reduce acute and late toxicities of the GI tract. Therefore, it becomes essential to provide successful treatment strategies in RT. To provide conformal dose distribution to large and complicated anatomical geometries such as WPRT (21–23), volumetric modulated arc therapy (VMAT) is an efficient dose delivery method.

Optimal segments to deliver conformal dose distributions are vital to implementing VMAT with high dose agreements successfully. Because MLC leaves move in one direction as the collimator angle is fixed, if the OAR are located between the separated target volumes along the same direction with the MLC movement, suboptimal MLC segments can be created. When each discretized gantry angle has one MLC segment, the limit of a maximum MLC traveling distance and speed between control points can restrict full utilization of the optimization engine’s capability to provide optimal MLC sequence (24–26). For example, while one side of the MLC conforms to half of the target contour, the other side cannot properly shield OAR or opens more than necessary outside of the planning target volume (PTV) to meet the prescribed dose. Thus, when the VMAT plan is optimized to cover a large and complex-shaped target, such as in WPRT, suboptimal MLC segments can be created and affect dose conformity.

In this study, VMAT optimized using a half-beam technique (VMAT-HF) was devised to provide superior OAR dose sparing, especially for the GI tract, and achieve dose conformity for the PTV in WPRT. The potential dosimetric benefits of the VMAT-HF were evaluated with dose homogeneity and conformity, dose–volume parameters, and normal tissue complication probability (NTCP) with statistical analysis. Compared to the dose distribution in VMAT optimized using a fully opened field (FF) to sufficiently cover the PTV, clinical usefulness of the VMAT-HF was suggested for dosimetric benefits and beam delivery with precise MLC leaf position accuracy.

## Materials and Methods

### Patient Selection

A total of 15 eligible patients diagnosed with anal, vaginal, and cervical cancer were included in this study on WPRT as per the National Comprehensive Cancer Network guidelines (27, 28). The patient and tumor characteristics are presented in **Table 1** with the treatment regimen. In patients with cervical and vaginal cancer, WPRT was followed by high-dose rate brachytherapy. All patients underwent computed tomography simulation in the supine position with arms on the chest. This retrospective dosimetric study was approved by the institutional review board of the Dongguk University Medical Center (110757-201711-HR-02-01), and written informed consent was obtained from all patients. All information was anonymized prior to analysis.

**Table 1:** Characteristics of patients, tumors, and treatment regimen selected for whole pelvic radiation therapy.

	Gender/Age	Origin	Stage	Pathology	Treatment Aim	WPRT-Prescribed Dose (Gy)	Chemotherapy
1	M/59	Anus	T2N0	SCC	Postoperative	50	–
2	F/75	Anus	Recurrent	SCC	Postoperative	50	–
3	F/78	Anus	T2N1	SCC	Definitive	50	FMC
4	M/88	Anus	T2N0	SCC	Definitive	41.4	FMC
5	M/78	Anus	T2M0	SCC	Definitive	54	–
6	F/51	Vagina	T2N0	AC	Definitive	45	–
7	F/61	Vagina	T1N0	SCC	Definitive	45	–
8	F/46	Vagina	T4N0	SCC	Definitive	45	WC
9	F/67	Cervix	T3aN0	SCC	Definitive	45	WC
10	F/71	Cervix	T3bN0	SCC	Definitive	45	WC
11	F/89	Cervix	T3aN0	AC	Definitive	45	–
12	F/86	Cervix	T3aN1	SCC	Definitive	45	–
13	F/87	Cervix	T3aN1	SCC	Definitive	45	–
14	F/79	Cervix	T4N1	SCC	Definitive	45	WC
15	F/77	Cervix	T4N1	SCC	Definitive	45	–

WPRT, whole pelvis radiotherapy; SCC, squamous cell carcinoma; FMC, 5-fluorouracil, mitomycin C; AC, adenocarcinoma; WC, weekly cisplatin.

### Target Delineation and Volumetric Modulated Arc Therapy Optimization Using Different Field Sizes

Clinical target volume and pelvic lymph nodes were delineated according to the consensus guidelines (29–31). The PTV was created by adding a 5-mm margin to the clinical target volume. Obturator, presacral, and internal iliac node chains are included in the pelvic lymph nodes in all cases. Treatment volume for patients with gynecological cancer includes the tumor involving the lower third of the vagina and tumor bed, parametrium, uterosacral ligaments, and pelvic lymph nodes. The small bowel, bladder, rectum, and femur heads were contoured as OAR. The anorectum in patients with anal cancer was contoured superiorly from the rectosigmoid flexure to the inferior level, 3 cm above the anal verge. Rectum was defined as rectum_wPTV_ or rectum_woPTV_ depending on whether it overlaps with PTV to avoid optimization conflicts between target dose coverage and rectal dose sparing. Each PTV received a prescribed dose of 50 or 46 Gy in 25 or 23 fractions as in **Table 1** except for one case. External iliac and perirectal nodes are included in patients with gynecological and anal cancer, respectively. For effective plan optimization to achieve conformal dose distributions, three pseudo structures are used, which are created by subtracting expanded PTVs with a 4-mm, an 8-mm, and a 14-mm margin from the body limited in the calculation volume.

**Figure 1:**
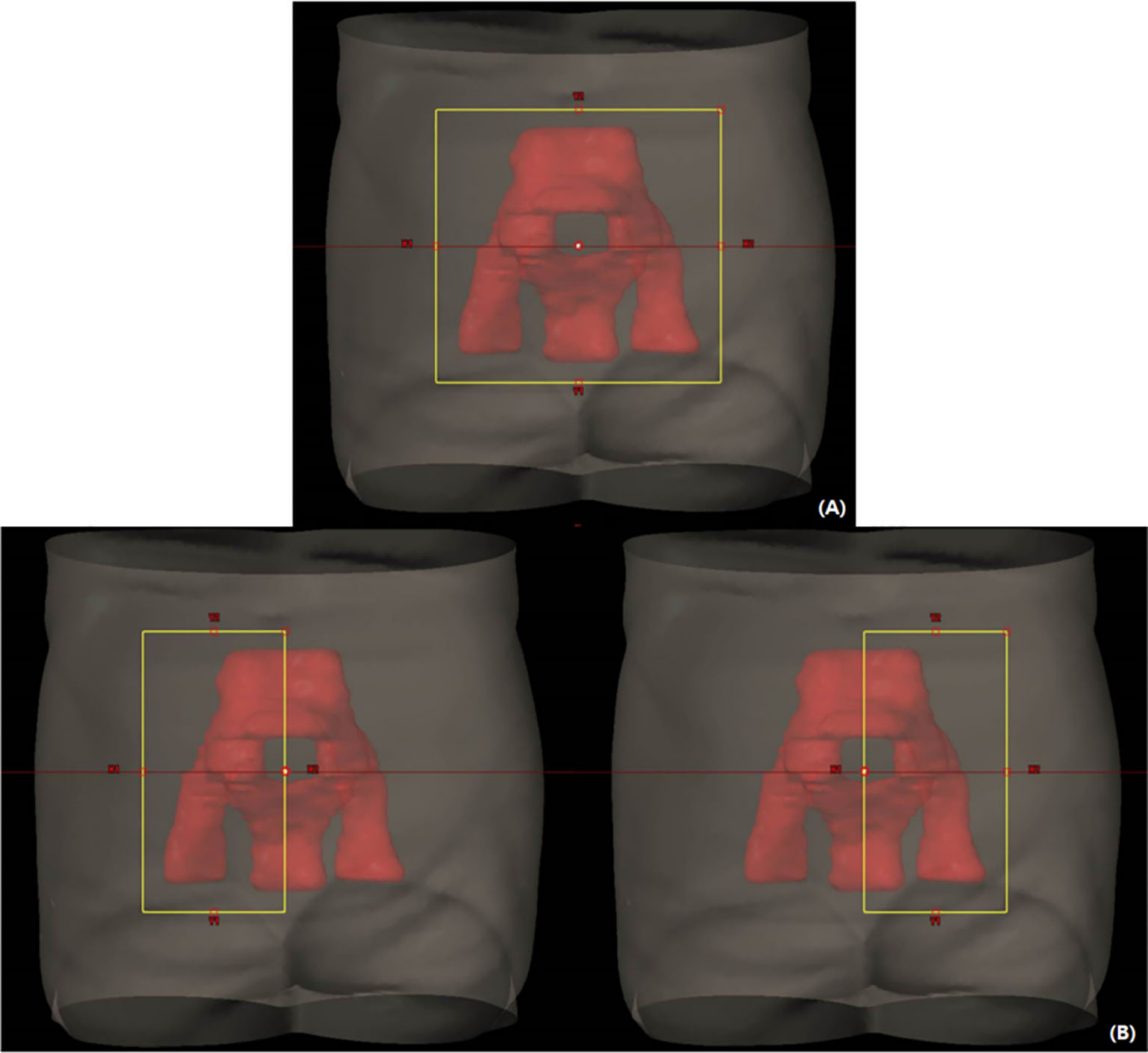
Volumetric modulated arc therapy (VMAT) plans optimized with different field sizes for whole pelvic radiation therapy. **(A)** A fully opened field size (FF) to sufficiently cover a planning target volume in a beam’s eye view across arc angles. **(B)** Half of the FF size for each arc rotating clockwise and counterclockwise.

All VMAT plans consisted of four full arcs alternating clockwise and counterclockwise in Eclipse (version 10.0, Varian Medical Systems, Palo Alto, CA). The collimator angles < ± 40° were used for each arc to improve dose conformity and minimize the interleaf leakage and tongue-and-groove effect in VMAT (32). Meanwhile, dose distributions of the VMAT plans were optimized using different field sizes, as shown in **Figure 1**. An optimized VMAT-FF is created with an FF sufficient to cover an entire PTV but <15 cm with X-jaws considering the maximum leaf span of the Millennium MLC (Varian Medical Systems). The opening of the FF was adjusted in a superior–inferior direction to sufficiently include the PTV plus a margin <1 cm, as shown in **Figure 1A**. Appropriateness of the field size was reviewed at all beam’s eye views at the different angles composed of VMAT arcs. The VMAT-HF is optimized with half the size of the FF. The optimized dose was delivered by opening the half and the other half of the FF for two arcs rotating clockwise and counterclockwise, respectively, as shown in **Figure 1B**.

The dose constraints were applied to meet dose criteria for target and OAR as per the radiation therapy oncology group protocols for each treatment site (1, 2). More specifically, the optimization objectives and their priorities for the structures were applied as shown in **Table 2** with automatic normal tissue optimization with a priority of 350. The dose distributions for each treatment site were calculated in Eclipse using Analytical Anisotropic Algorithm (version 10.0, Varian Medical Systems) and progressive resolution optimizer (version 10.0, Varian Medical Systems). The dose was calculated with a grid size of 2.5 mm. Dosimetric benefits of VMAT-FF and VMAT-HF were compared when the prescribed dose covered 95% of the PTV with the same dose constraints applied for OAR.

**Table 2:** Plan optimization objectives for target and organs at risk (OAR) using dose–volume parameters and relative weights, when 95% of the planning target volume is covered by the prescribed dose (R_p_) in volumetric modulated arc therapy (VMAT) plans.

Structure			Dose–volume objectives	Relative weight
Target	CTV		D_max_ < (R_p_ × 1.03) Gy	500
			D_100%_ > R_p_	400
	PTV		D_max_ < (R_p_ × 1.03) Gy	500
			D_100%_ > (R_p_ × 0.98) Gy	400
OAR	Bladder		D_max_ < (R_p_ × 1.03) Gy	350
			D_30%_ < 30 Gy	170
			D_50%_ < 25 Gy	170
	Colon		D_max_ < (R_p_ × 1.03) Gy	350
			D_30%_ < 33 Gy	165
			D_50%_ < 28 Gy	165
	Small bowel		D_max_ < (R_p_ × 1.03) Gy	350
			D_15%_ < 40 Gy	180
			D_30%_ < 33 Gy	180
			D_50%_ < 26 Gy	180
			D_75%_ < 20 Gy	180
	Rectum	Rectum_woPTV_	D_max_ < (R_p_ × 1.03) Gy	350
			D_30%_ < 30 Gy	175
			D_50%_ < 25 Gy	175
		Rectum_wPTV_	D_max_ < (R_p_ × 1.03) Gy	350
			D_60%_ < 48 Gy	170
			D_80%_ < 46 Gy	170
	Right or left femoral head		D_max_ < (R_p_ × 1.03) Gy	350
			D_50_ < 35 Gy	160
			D_30_ < 40 Gy	160
	Body		D_max_ < (R_p_ × 1.05) Gy	600

D_max_, maximum point dose; D_volume%_, dose received by % of the structure volume; rectum_woPTV_, a case where the rectum is overlapped with a planning target volume; rectum_wPTV_, a case where the rectum is not overlapped with a planning target volume.

### Beam Modulation Complexity

The traveling distances and segment shapes between MLC control points can affect the complexity of the VMAT intensity modulation. The modulation complexity score (MCS), using variabilities of leaf sequences (LSV) and segment area (AAV), was adopted to comprehensively present the plan complexity across all segments (33). It is formulated using equation (1) by reflecting each segment weight to the corresponding relative arc weight.

(1) $$\text{MC}{\text{S}}_{\text{VMAT}}=\Sigma_{\text{arc}=1}^{\text{N}}\Sigma_{\text{cp}=1}^{\left(\text{n}-1\right)}\left[\left(\frac{\text{AA}{\text{V}}_{\text{cp}}^{\text{arc}}+\text{AA}{\text{V}}_{\text{cp}+1}^{\text{arc}}}{2}\right)\times \left(\frac{LS{\text{V}}_{\text{cp}}^{\text{arc}}+\text{LS}{\text{V}}_{\text{cp}+1}^{\text{arc}}}{2}\right)\times \left(\frac{\text{M}{\text{U}}_{\text{cp}+1}^{\text{arc}}-\text{M}{\text{U}}_{c\text{p}}^{\text{arc}}}{\text{M}{\text{U}}^{\text{arc}}}\right)\right].$$

The parameters of n and N indicate the total number of control points per arc and the total number of arcs used in each VMAT plan. The LSV_cp_ and AAV_cp_ for each control point are calculated using equations (2) and (3), respectively, where m is the number of MLC leaves that move underneath the unblocking portion of the field defined by X and Y jaws for each control point:

(2) $$LSV_{cp}=\frac{\Sigma_{i=1}^{m}\left(pos_{L}-\left|(pos_{i+1}^{L}-pos_{i}^{L}\right|\right)}{(m-1)\times pos_{L}^{cp}}\times \frac{\Sigma_{i=1}^{m}\left(pos_{R}-\left|(pos_{i+1}^{R}-pos_{i}^{R}\right|\right)}{(m-1)\times pos_{R}^{cp}}$$

(3) $$AAV_{cp}=\frac{\Sigma_{i=1}^{m}(pos_{i}^{L}-pos_{i}^{R})}{m(pos_{L}^{arc}-pos_{R}^{arc})}$$

The $pos_{i}^{L}$ presents the i-th leaf position of the MLC at the left bank. The $pos_{L}^{cp}$ and $pos_{L}^{arc}$ indicate the farthest position of the MLC leaf from the isocenter among all MLC leaves constituting a shape of the individual segment and across all control points of an individual arc. R denotes the MLC leaves on the right bank. The LSV presents variability of the MLC leaf traveling distances sweeping each set of control points relative to the maximum lateral separation from the isocenter for each side. The AAV presents the complexity of separation of each pair of MLC leaves relative to the maximum separation created among all MLC leaves across all segments consisting of the arc.

### Dose Evaluation for Target and Organs at Risk With Statistical Analysis

To compare dose distributions in two VMAT plans depending on MLC segments and sequences, dose conformity (CN) for PTV was calculated using equation (4). The TV is the target volume. The TV_RI_ and V_RI_ mean the target volume and the volume covered by the reference prescribed isodose, respectively (34). The ideal value of CN is 1. As it is closer to 1, the dose distribution is more conformal to the target. In addition, two different formulas were used to calculate dose homogeneity for the PTV. One is the homogeneity index (HI) proposed by ICRU-83 (35). The other is the s-index representing the standard deviation (D_SD_) of doses predicted to the PTV (36). The HI was calculated using doses for the 2% (D_2%_), 98% (D_98%_), and 50% (D_50%_) of the PTV, as shown in equation (5). The standard deviations of the dose element (D_i_) for each voxel volume (v_i_) of the PTV were calculated to the prescribed dose (D_Rp_) for the TV (36) using equation (6). The ideal value of HI and s-index is 0. The closer the value is to 0, the better the dose distribution is homogeneous to the prescribed dose.

(4) $$\text{CN}=\frac{TV_{RI}}{TV}\times \frac{TV_{RI}}{V_{RI}}$$

(5) $$\text{HI}=\frac{D_{2\%}-D_{98\%}}{D_{50\%}}$$

(6) $$s-index=D_{SD}=\sqrt{{\sum \left[\left(\frac{D_{i}-D_{Rp}}{D_{Rp}}\right)\times 100\right]}^{2}\times \frac{v_{i}}{TV}}$$

Dose sparing for OAR was evaluated with dose–volume histograms (DVHs) and dose–volume parameters associated with acute and late toxicities. Since the dose–volume predictors on acute or late GI toxicities can be different depending on patient surgery, concurrent therapy, prescribed dose, and treatment techniques of RT (18, 37–44), each volume receiving the doses from 5 to 45 Gy was evaluated for small bowel and colon, with a dose interval of 5 Gy. Maximum (D_max_) and mean (D_mean_) dose and the dose (D_2cc_) delivered to the 2 cc of organ volume were also analyzed. Radiobiological effects were estimated by calculating the equivalent uniform dose (EUD) and NTCP using Emami–Burman parameters (45). To present the sensitivities and variabilities of NTCP in terms of analytic models, Lyman–Kutcher–Burman and EUD-based log-logistic models were also adopted (46, 47). The alpha–beta ratios for acute and late toxicities and required biological parameters to calculate NTCP are shown in **Table 3** (43, 46–48). Because volume variations at doses can be different for rectum_wPTV_ and rectum_woPTV_, DVH was separately presented in anal cancer cases. Both rectum_wPTV_ and rectum_woPTV_ were combined to evaluate dose–volume parameters and NTCP. Furthermore, to distinguish the statistically significant OAR dose sparing and the consequent effect of VMAT-HF from VMAT-FF, the Wilcoxon signed-rank test was performed using statistical analysis software (SPSS version 20, SPSS Inc., Chicago, IL). A p-value <0.05 was considered statistically significant.

**Table 3:** Radiobiological parameters to calculate equivalent uniform dose and normal tissue complication probabilities for the bladder and gastrointestinal tract at different endpoints and alpha–beta ratios.

OAR	α/β [Gy]	Endpoint	Parameters for LKB model		Parameter for log-logistic model	TD_50_
			n	m	γ	
Bladder	5	Late reaction	0.5	0.11	3.63	80
	7.5	Shrinkage/Ulceration			
	10	Acute cystitis			
Small intestine	4	Ulcer/Obstruction	0.15	0.16	2.49	55
	8	Acute malabsorption			
Colon	10	Early reactions	0.17	0.11	3.63	55
Rectum	2.5	Late reactions	0.09	0.13	3.07	76.9
	5.4	Chronic inflammation/Ulcer			

LKB, Lyman–Kutcher–Burman.

### Plan Deliverability

The VMAT plan’s deliverability was evaluated by generating Dyanlog files through dry runs for each patient case (49). As the sensitivity and accuracy of the MLC position errors are significant in dose agreement in IMRT (50), MLC position errors of the VMAT-FF and VMAT-HF were investigated with the log information recorded every 50 ms. The beam-on times required to deliver different monitor units (MUs) were compared between VMAT-FF and VMAT-HF. In addition, the positions of each MLC leaf, which moves inside the field defined by the jaw, were compared with the corresponding planned MLC positions to analyze errors at individual control points. Data in the log files were analyzed in a customized code written by Matlab (version 9.0.0.96032; The MathWorks, Inc., Natick, MA).

## Results

**Figure 2:**
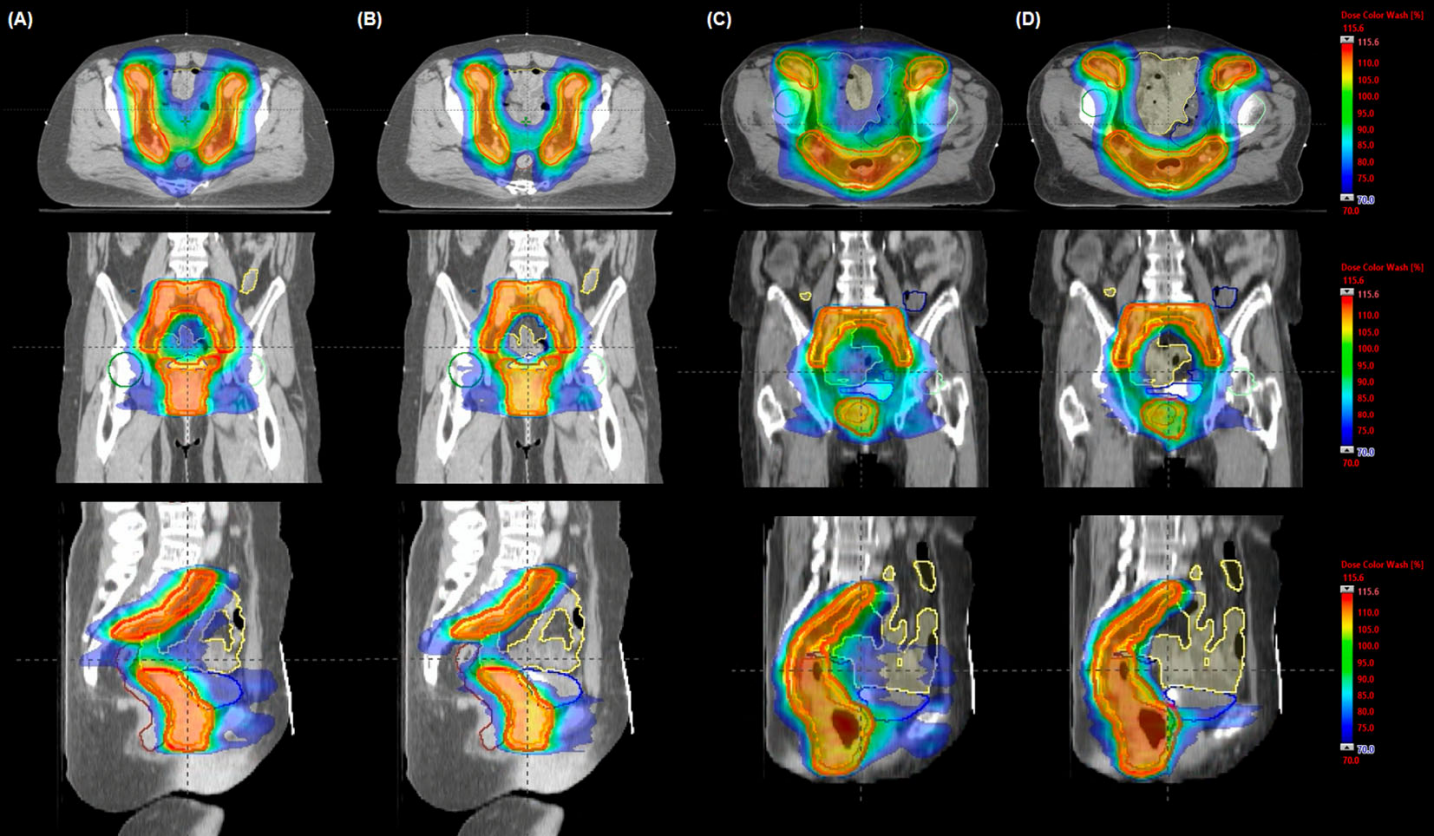
Comparison of representative dose distributions in volumetric modulated arc therapy (VMAT) plans for a cervix **(A, B)** and an anal **(C, D)** cancer case. The dose was optimized using **(A, C)** a fully opened field size (VMAT-FF) to cover the planning target volume and **(B, D)** a half-beam technique (VMAT-HF). Isodose and structures displayed as a color-wash overlay and delineated contours, respectively: red for planning target volume, yellow for small bowel, navy for colon, brown for anorectum, blue for bladder, green for right femur head, and cyan for left femur head.

**Figure 3:**
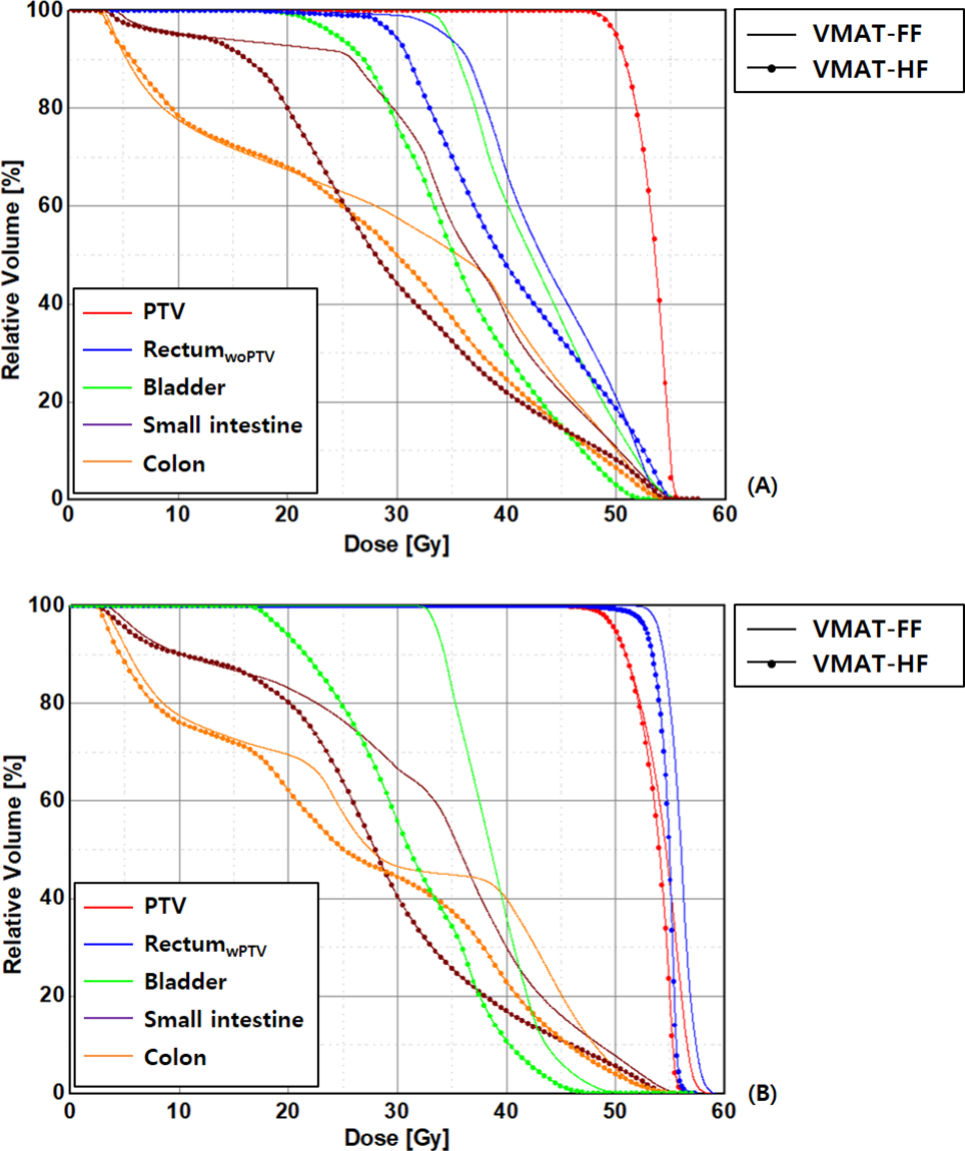
Comparison of dose–volume histograms in patients with anal cancer in the volumetric modulated arc therapy (VMAT) plans optimized using a fully opened field size (FF) and a half-beam technique (HF), as rectal volume is **(A)** overlapped (rectum_wPTV_) and **(B)** not overlapped (rectum_woPTV_) with planning target volumes.

As dose distributions of each VMAT plan are compared, VMAT-HF showed that a 70%–75% isodose line compactly surrounds the target shapes. As **Figure 2** shows superior high dose gradients in a representative cervix and an anal cancer case, VMAT-HF resulted in sculpted dose curvatures along the posterior bladder wall and anterior rectal wall. It shows conformal dose distribution for the target in the axial and the sagittal view (the first and the third rows in **Figure 2**). Such dose sparing for the bladder is also distinguishable in the coronal view (the second row in **Figure 2**). The VMAT-HF demonstrates the isodose distribution of less than 70% between the left and right iliac lymph nodes. The OAR dose sparing in VMAT-HF was manifested in the DVH comparisons, as shown in **Figure 3**. As the DVHs for the PTV were identical in two VMAT plans, the small intestine and colon showed noticeable dose reduction at the intermediate dose range from 25 to 45 Gy, as in **Figure 3A**. The volume reduction was up to 35% at 25–30 Gy and 15% at 35–40 Gy for the small intestine and colon. The volume reduction at the dose ranges from 30 to 50 Gy was also observed in rectum_woPTV_. The VMAT-HF achieved the sharper dose fall-off for the PTV and rectum_wPTV_ without an excessive hot spot, as shown in **Figure 3B**. The bladder DVH showed a noticeable dose–volume difference from 20 to 50 Gy in VMAT-HF.

The differences of OAR dose sparing were more presented explicitly in **Table 4** with major dose–volume parameters, which showed statistical significance. The reduction of intermediate or high dose to OAR in VMAT-HF led to reducing D_mean_. The VMAT-HF showed volume reduction at the dose range from 20 to 45 Gy for small bowel, colon, and bladder. The small bowel and bladder showed significant volume reduction at 15 Gy as well. The rectum also showed dose–volume sparing at the dose range from 30 to 45 Gy. Furthermore, the VMAT-HF resulted in significantly lower EUD and NTCP in LKB and logistic models for the small bowel and colon, as in **Table 5**.

**Table 4:** Comparison of dose–volume parameters for the small bowel, colon, rectum, and bladder in the volumetric modulated arc therapy (VMAT) plans optimized using a fully opened field (FF) and a half-beam technique (HF) with statistical analysis using Wilcoxon signed-rank test.

OAR		VMAT-FF	VMAT-HF	p-value
Small bowel	D_mean_ [Gy]	32.1 ± 3.9	29.3 ± 2.6	0.001
	V_45Gy_ [%]	17.0 ± 7.6	13.2 ± 5.1	0.002
	V_30Gy_ [%]	58.8 ± 15.8	43.0 ± 12.7	0.001
	V_15Gy_ [%]	89.4 ± 5.4	88.4 ± 5.0	0.012
Colon	D_mean_ [Gy]	26.3 ± 6.4	24.4 ± 5.8	0.001
	D_2cc_ [Gy]	53.6 ± 2.2	52.7 ± 2.4	0.008
	V_45Gy_ [%]	15.8 ± 7.0	12.2 ± 6.3	0.001
	V_30Gy_ [%]	41.0 ± 20.1	34.4 ± 15.7	0.012
Rectum	D_mean_ [Gy]	45.2 ± 7.4	44.2 ± 7.8	0.003
	V_45Gy_ [%]	56.4 ± 33.6	53.1 ± 34.6	0.003
	V_30Gy_ [%]	92.8 ± 9.4	88.5 ± 11.6	0.003
Bladder	D_mean_ [Gy]	38.9 ± 2.7	34.7 ± 3.9	0.001
	D_2cc_ [Gy]	52.0 ± 2.3	51.0 ± 2.8	0.006
	V_45Gy_ [%]	22.4 ± 10.4	16.6 ± 8.3	0.008
	V_30Gy_ [%]	90.7 ± 11.7	69.5 ± 21.4	0.005
	V_15Gy_ [%]	100	98.4 ± 4.2	0.043

**Table 5:** Comparison of normal tissue complication probabilities (NTCP) using Lyman–Kutcher–Burman (LKB) and logistic models and equivalent uniform dose (EUD) with statistical analysis using Wilcoxon signed-rank test in volumetric modulated arc therapy (VMAT) plans.

Organ at Risk		VMAT-FF	VMAT-HF	p-value^*^
	LKB [%]	11.74 ± 5.52	8.61 ± 3.69	0.001
Small bowel	Logistic [%]	10.98 ± 5.49	7.80 ± 3.57	0.001
	EUD [Gy]	39.91 ± 2.99	38.57 ± 2.43	0.001
	LKB [%]	3.01 ± 2.36	1.69 ± 1.64	0.001
Colon	Logistic [%]	3.08 ± 2.32	1.78 ± 1.62	0.001
	EUD [Gy]	38.37 ± 3.00	36.71 ± 3.02	0.001

Values are presented as mean ± standard deviation.

VMAT-FF, VMAT plan optimized using a fully opened field; VMAT-HF, VMAT plan optimized using a half-beam technique.

*Comparison of VMAT-FF and VMAT-HF.

**Figure 4:**
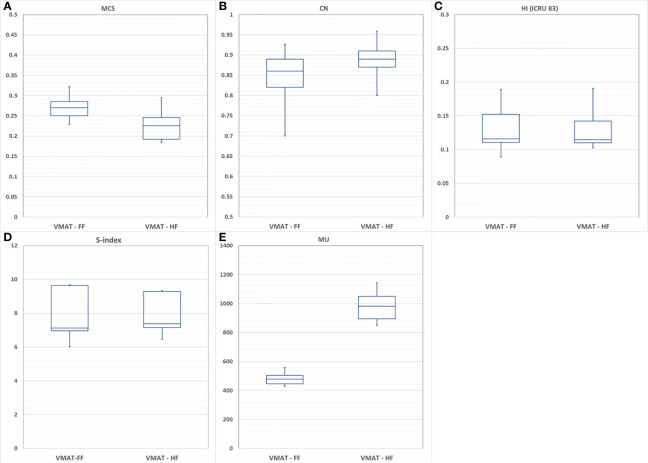
Comparison of volumetric modulated arc therapy (VMAT) optimized with using a fully opened field (VMAT-FF) and a half-beam technique (VMAT-HF) with **(A)** modulation complexity score (MCS), **(B)** conformity number (CN), **(C)** homogeneity index (HI), **(D)** s-index, and **(E)** monitor units (MU).

When the dosimetric benefits were analyzed in terms of MCS, the VMAT-HF showed that it used 17% less modulation complexity as in **Figure 4A** but achieved superior dose conformity (0.89 *vs*. 0.85 in average) as in **Figure 4B**. The VMAT-HF as compared to VMAT-FF showed a significant difference in AAV (0.29 ± 0.04 *vs*. 0.35 ± 0.04, p-value: 0.001) and LSV (0.71 ± 0.02 *vs*. 0.79 ± 0.01, p-value: 0.001). Both plans showed comparable dose homogeneity with HI and s-index in **Figures 4C, D**. In addition, as shown in **Figure 4E**, while VMAT-HF used two times higher MU than VMAT-FF, beam-on time was identical for both plans because VMAT can adjust dose-rate in beam delivery. MLC leaf position average errors were also comparable between VMAT-HF and VMAT-FF (0.36 ± 0.40 mm *vs*. 0.39 ± 0.36 mm, p-value: 0.013).

## Discussion

The HF has been used in the field matching for head and neck cancer and breast cancer when supraclavicular lymph nodes are included in the treatment volume (51, 52). A mono-isocentric technique using the HF facilitated a more reliable patient setup and simple beam matching using a non-divergent beam edge. In addition, the HF at the matching line could bring out dose reduction of the lung in breast treatment. Maintaining such dosimetric benefit and more effectively reducing doses to OAR, studies were expanded to employ intensity modulation using optimal segments from HF (26, 53, 54). Consequently, VMAT-HF showed reducing volume receiving a dose of less than 10 Gy for left-sided breast cancer, which is more challenging than the right-sided case due to heart dose sparing. Furthermore, the VMAT using a fixed-jaw (opening of 15 cm in the X direction), considering the limitation of maximum leaf span of the MLC, also showed parotid dose sparing (26).

However, the HF has not been used for WPRT because the whole pelvic region is not usually regarded as a site that needs beam matching or can be covered with the half size of the FF for a large PTV. If we can make the most use of the capability of an optimization engine to generate optimal fluence and leaf sequencing, dose optimization using the HF and multiple arcs can effectively meet plan objectives in VMAT. In light of using optimal MLC segments, the VMAT-HF could demonstrate dosimetric benefits for WPRT with superior dose conformity and effective OAR sparing while using less beam modulation complexity.

**Figure 5:**
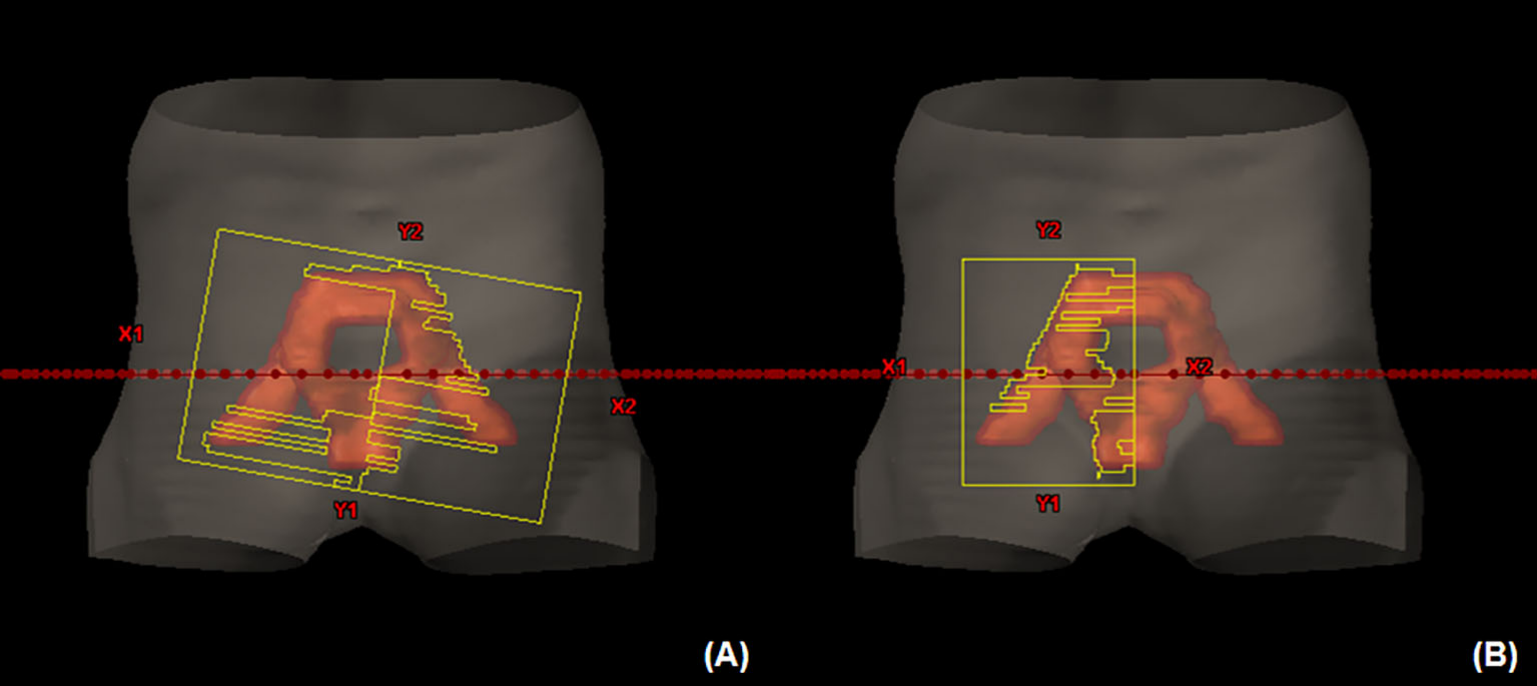
**(A)** A suboptimal multi-leaf collimator (MLC) segment in volumetric modulated arc therapy (VMAT) optimized using a fully opened field compared to **(B)** an MLC segment for the planning target volume in VMAT optimized using a half-beam technique at the beam’s eye view.

To cover the large PTV with the prescribed dose, especially when the fixed field size is required before starting dose optimization in treatment planning, opening a sufficient field size covering the PTV plus a margin is typically considered. However, when the FF is used to treat complex-shaped PTV, particularly having some separated subvolumes as shown in **Figure 5A**, suboptimal segments or non-blocking areas can be created. Although the optimization engine tries to spare doses to the normal tissues between the subvolumes, it can conflict with PTV dose coverage. However, MLC is limited to move along one direction, and only one MLC segment is allowed at one discretized gantry angle in VMAT. Then, the MLC segment may have to adopt unnecessary dose delivery to normal tissues. Consequently, it can deteriorate dose conformity. Even if more stringent OAR constraints are applied to improve the quality of dose optimization in VMAT-FF, it could be challenging to improve dose conformity without increasing hot spots unless the MLC segments are improved. However, HF can effectively guide the optimization engine to utilize its ability for a limited area. As the HF is integrated with VMAT, which uses arc beams going through different gantry angles, VMAT-HF can successfully induce optimal and deliverable MLC segments, as shown in **Figure 5B**.

Such an optimal MLC segment in VMAT-HF led to achieving comparable or superior conformal dose distributions without excessive beam modulation presented with MCS. The modulation index can be a useful tool to comprehensively evaluate beam modulation complexities based on MLC segments (55). However, the lower modulation complexity and indices would not always result in precise dose delivery showing better agreement between predicted and delivered doses (56, 57). In this study, without sacrificing beam delivery efficiency and accuracy in terms of beam-on time and MLC position accuracy, VMAT-HF could achieve effective OAR dose sparing.

Different dose–volume parameters were used to find the best predictor to reduce the incidence of acute and late GI toxicities. The GI tract showed radiation dose–volume effects and maximum dose effect to a small volume in estimating acute and late toxicity. Clinical studies showed significant dose–volume predictors on acute and late GI toxicities according to treatment modalities before and after RT, prescribed doses, and dose fractionations (58, 59). In WPRT using IMRT, while the volume receiving high-dose >45 Gy showed a strong correlation with a higher incidence of acute small bowel’s toxicity for gynecologic cancers (14), less acute GI toxicity was observed by a reduction of volume receiving the intermediate dose of 30–35 Gy among prostate patients receiving a whole pelvic dose of 54 Gy (40). Three-dimensional conformal RT for rectal cancer showed a significant correlation of acute toxicity with the volume exposed to a lower dose, particularly at or less than 15 Gy, for small bowel during chemoradiotherapy (44). When the maximum dose in the small bowel or colon has to be compromised for target dose coverage, volume reduction to high and intermediate dose could reduce GI tract toxicities (38, 40). As VMAT-HF showed statistically significant GI and GU dose sparing to intermediate-dose >15 Gy and lower NTCP compared to VMAT-FF, potentially less acute and late GI toxicity can be likely expected in WPRT using VMAT-HF.

## Conclusions

This dosimetric study was conducted to effectively save OAR doses for WPRT *via* dose optimization using the HF. The VMAT-HF achieved noticeable physical dose sparing for GI tract and bladder and significantly lower NTCP even using less beam modulation complexity. The VMAT-HF showed conformal dose distribution using optimal MLC segments without non-blocking phenomena. The VMAT-HF showed potential dosimetric benefits compared to VMAT-FF without sacrificing beam delivery efficiency and MLC leaf position precision.

## Data Availability Statement

The original contributions presented in the study are included in the article/supplementary material. Further inquiries can be directed to the corresponding authors.

## Ethics Statement

This retrospective dosimetric study was approved by the Institutional Review Board of the Dongguk University Medical Center (110757-201711-HR-02-01), and written informed consent was obtained from all patients.

## Author Contributions

HJ, JP, MA, and H-JP conceived this study. HJ collected clinical cases and delineated structures for VMAT. JP, MA, YZ, and H-JP performed treatment planning. JP, MA, and YZ created computation modules to analyze results. JR analyzed the data and conducted the statistical analysis. SH, PJ, M-HK, MC, Y-TO, and ON provided clinical expertise and reviewed the VMAT plans and data. All authors contributed to the article and approved the submitted version.

## Conflict of Interest

The authors declare that the research was conducted in the absence of any commercial or financial relationships that could be construed as a potential conflict of interest.

## Publisher’s Note

All claims expressed in this article are solely those of the authors and do not necessarily represent those of their affiliated organizations, or those of the publisher, the editors and the reviewers. Any product that may be evaluated in this article, or claim that may be made by its manufacturer, is not guaranteed or endorsed by the publisher.
